# Kainic Acid-Induced Golgi Complex Fragmentation/Dispersal Shifts the Proteolysis of Reelin in Primary Rat Neuronal Cells: An In Vitro Model of Early Stage Epilepsy

**DOI:** 10.1007/s12035-015-9126-1

**Published:** 2015-03-21

**Authors:** Yuji Kaneko, Robert Sullivan, Travis Dailey, Fernando L. Vale, Naoki Tajiri, Cesar V. Borlongan

**Affiliations:** Center of Excellence for Aging and Brain, Department of Neurosurgery and Brain Repair, University of South Florida College of Medicine, 12901 Bruce B Downs Blvd, Tampa, FL 33612 USA

**Keywords:** Initiating epilepsy, Kainic acid, Glycosylation, Primary rat neuronal cell, Golgi protein

## Abstract

The endoplasmic reticulum-lysosome-Golgi network plays an important role in Reelin glycosylation and its proteolytic processing. Golgi complex fragmentation is associated with the separation of Reelin from this network. Kainic acid (KA) is an excitotoxic agent commonly used to induce epilepsy in rodents. The relationship between KA-induced neuronal damage and Golgi complex fragmentation has not been investigated, leaving a major gap in our understanding of the molecular mechanism underlying the development of pathophysiology in epilepsy. We cultured primary rat cortical neurons eitherin ambient condition (control) or treated with a range of KA doses to reveal whether Golgi complex fragmentation impaired neuronal function. The half-life maximal inhibitory concentration (*IC*_50_) value of KA was detected to be approximately 5 μM, whereby at these concentrations, KA impaired neuronal viability, which was closely associated with initial Golgi complex fragmentation and subsequent reduction in both the expression and glycosylation patterns of Reelin. These findings implicate that Golgi complex fragmentation and Reelin dysfunction are key contributors to neuronal cell death in the early stage of epilepsy pathophysiology, thereby representing as novel disease biomarkers, as well as potent therapeutic targets for epilepsy.

## Introduction

Epilepsy is a debilitating neurological disorder affecting approximately 2 % of the world’s population. Over three million Americans suffer from some form of epilepsy, with mesial temporal lobe epilepsy (TLE) being the most common, arising from the temporal lobe structures of amygdala, hippocampus, and parahippocampal gyrus [1]. Despite the availability of many antiepileptic drugs (AEDs), greater than 30 % of TLE patients will have seizures refractory to standard therapeutics, highlighting the need for more effective therapies. [2].

The Golgi complex is localized around the centriole, a regulator of cell-cycle progression, and is a cytoplasmic organelle that participates in maturation and modification of membrane proteins during transport from the site of synthesis in the endoplasmic reticulum (ER) [3, 4]. Golgi complex fragmentation irreversibly occurs during apoptosis, when the Golgi complex fragments to produce clusters of vesicles that disperse throughout the cytoplasm [5]. The Golgi complex fragmentation not only is essential for cell entry into mitosis [6] but also is a pathological feature of several neurodegenerative diseases including Alzheimer’s disease [7], Parkinson’s disease [8], amyotrophic lateral sclerosis [9], and Creutzfeldt-Jakob disease [10].

Reelin is an extracellular matrix glycoprotein [11] synthesized and secreted by Cajal-Retzius cells in the marginal zone of the cortex [12]. It localizes to Golgi complex [13] where it is extensively processed within the ER-lysosome-Golgi network [5]. Secreted Reelin binds the apolipoprotein 2 receptor (ApoE2R) and the very low density lipoprotein receptor (VLDLR) [14] as well as the extracellular domain of ephrin B proteins (EFNBs) [15], which associate with ApoE2R and VLDLR. These interactions in turn induce phosphorylation of the cytoplasmic adaptor protein Disabled-1 (Dab-1) by Src family tyrosine kinases (SFKs), Fyn and Src, associated with signaling of integrin pathways [16]. The physiological dysfunction of Reelin has been implicated in the pathogenesis of several neurodegenerative diseases including epilepsy[17, 18], depression [19], schizophrenia [15], and Alzheimer’s disease [11, 20]. The ER-lysosome-Golgi network assumes a major function in Reelin protein modification (glycosylation), its proteolytic processing (180- and 320-kDa fragments), and the synthesis of proteoglycans, which are composed of molecules of extracellular matrix [11].

Unilateral injection of kainic acid (KA), an agonist to the excitatory neurotransmitter glutamate, into rodent brain induces epileptic activity and epileptic seizures, and granule cell dispersion [21]. KA possesses neurotoxicity involving epileptogenic effect via the α-amino-3-hydroxy-5-methyl-4-isoxazolepropionic acid (AMPA) and kainate receptors that respond to the neurotransmitter glutamate [22]. KA-induced caspase activation and subsequent DNA fragmentation are key features of the apoptotic pathway leading to neuronal death [23, 24]. However, KA-induced Golgi complex fragmentation remains to be examined in electrophysiological hyperactivity models, which mimic seizure and epilepsy.

In this study, we observed that the administration of KA in primary rat neuronal cells promoted neuronal dysfunction by facilitating the Golgi complex fragmentation/dispersal, which in turn may damage a sophisticated network of biochemical reactions involved in energy production and macromolecular biosynthesis. The present results demonstrated that a dysfunctional Golgi-Reelin interaction may contribute to the disease pathophysiology of epilepsy.

## Materials and Methods

### Cell Culture

Primary rat neuronal cells (PRNCs) were obtained from BrainBit (E18 rat cortex). According to the previous reference[25], cells (4 × 10^4^ cells/well) were suspended in 200 μl Neural Medium (NbActive 4, BrainBit NB4-500) containing 2 mM l-glutamine and 2 % B27 in the absence of antibiotics and grown in poly-l-lysine-coated 96-well plates (BD 354516) at 37 °C in humidified atmosphere containing 5 % CO_2_ in 40 % of the neuron and 60 % astrocyte cell population [25]. After 3 days of culturing (approximately 60 % cell confluence), PRNCs were exposed to KA (1.25 nM ~ 125 μM) for 3 days at 37 °C.

### Measurement of Cell Viability

Measurement of cell viability was performed by both fluorescent live/dead cell assay and trypan blue exclusion method [26]. A two-color fluorescence cell viability assay was performed by calcein acetoxymethyl ester (calcein-AM, Invitrogen C3099) to be retained within live cells, including an intense uniform green fluorescence and ethidium homodimer (EthD-1) to bind the nuclei of damaged cells. Following treatment, the cells were incubated with 2 μM calcein-AM and 4 μM EthD-1 for 45 min at room temperature in the dark. After washing once with phosphate-buffered saline (PBS) (Sigma-Aldrich P-3813), the green fluorescence of the live cells was measured by the Gemini EX florescence plate reader (Molecular Device), excitation at 490 nm and emission at 520 nm. In addition, trypan blue (Gibco 15250, 0.4 %) exclusion method was conducted, and mean viable cell counts were calculated in four randomly selected areas (1 mm^2^, *n* = 10) to reveal the cell viability. Briefly, within 5 min after adding trypan blue, we digitally captured under microscope (×200) ten pictures (approximately 100 cells/picture) for each condition, then randomly selected five pictures, and counted the number of cells for each individual treatment condition. Normalized cell viability was calculated from the following equation: viable cells (%) = [1.00 – (number of blue cells / number of total cells)] × 100. To precisely calibrate the cell viability, the values were standardized from florescence intensity and trypan blue data [25].

### Measurement of Mitochondrial Activity

Following cell culture, reduction of 3-(4,5-dimethyl-2-thiazoyl)-2,5-diphenyltetrazolium bromide (MTT) (Roche 11465007001) by mitochondrial dehydrogenases was used as a measure of mitochondrial activity as previously described [25]. Briefly, the cells were treated with 0.5 mg/ml MTT solution at 37 °C for 4 h and subsequently incubated with lysis buffer for 14 h in the incubator in a humidified atmosphere at 37 °C and 5 % CO_2_. The optical density of solubilized purple forzmazan was measured at 570 nm on a Synergy HT plate reader (Bio-Tex).

### Western Blot Analysis

PRNCs were treated with CelLytic MT mammalian lysis reagent (Sigma-Aldrich, C3228) with protease inhibitor cocktail (Sigma-Aldrich, I3786). The lysate was centrifuged at 3000 *g* and 4 °C for 15 min, and the supernatant was stored at −80 °C until used. Protein samples (10 ~ 60 μg/lane) were run on 4 ~ 14 % Tris-Glycine SDS-PAGE gel [27] and then transferred onto a nitrocellulose membrane (Bio-Rad 162-0112) at 30 V, 4 °C for 14 h. The nitrocellulose membranes were treated with PBS containing 0.1 % Tween-20 and 5 % non-fat milk (Bio-Rad 170-6404) for 45 min at room temperature. Membranes were then incubated with the primary antibodies, anti-Reelin mouse antibody (LSBio LS-C90872/49638, 1/500), anti-human natural killer-1 (HNK1) rabbit antibody (Bioss bs-1635R, 1/500), anti-glyceraldehyde-3-phosphate-dehydrogenase (GAPDH) [6C5] mouse antibody (Abcam 8245, 1/5,000), and rabbit polyclonal anti-*cis*-Golgi glycoprotein of 130 kDa (GM130, Abcam ab28049, 1/500), at 4 °C for 14 h. After washing with PBS containing 0.1 % Tween-20 (PBST), the nitrocellulose membrane was incubated with donkey anti-mouse IRDye800® CW secondary antibody (LI-COR 926-32212, 1/5000) or donkey anti-rabbit IRDye800®CW secondary antibody (LI-COR 926-32213, 1/5000) for 90 min at room temperature in the dark. Immunoreactive detection using near-infrared fluorescence was performed according to protocol of Odyssey® Infrared Imaging System (LI-COR®). Molecular weight estimation of the full-length Reelin protein (400 kDa) and its fragments (180 and 320 kDa) using SeeBlue® Plus Pre-stained standard (Invitrogen, LC5925) was calculated from plotting a standard curve of logarithm molecular weight versus target protein mobility.

### IP Assays

After PRNC, whole cell lysate (400 μg/100 μl PBS containing protease inhibitors (Sigma-Aldrich, I3786)) was interacted with anti-Reelin mouse monoclonal antibody (LSBio LS-C90872/49638, 1/50) at 4 °C for 16 h, and the lysate was incubated with 50-μl Protein G agarose beads (Invitrogen 10-1241) at 4 °C for 4 h [11]. Following antibody-protein, G complexes were washed three times with 4 °C RIPA buffer (Sigma-Aldrich R0278), and immune complexes were recovered by heating the samples at 95 °C for 5 min in Laemmli SDS lysis buffer (Bioland Scientific LLC SAB02-01).

### Immunocytochemistry Analysis

After PRNCs (8 × 10^4^ cell/well) were cultured in 400-μl Neural medium containing 2 mM l-glutamine and 2 % B27 in the absence of antibiotics in poly-l-lysine 8-chamber (BD 354632) for 3 days, the cells were then exposed to 5 μM KA for 3 days and fixed in 4 % paraformaldehyde [25]. The cells were washed five times for 10 min in PBST. Then, they were blocked by 5 % normal goat serum (Invitrogen 50062Z) in PBST for 1 h at room temperature. Primary antibodies, mouse monoclonal anti-Golgi formiminotransferase cyclodeaminase (FTCD, Abcam ab27043, 1/400), rabbit polyclonal anti-*cis*-Golgi glycoprotein of 130 kDa (GM130, Abcam ab28049, 1/400), rabbit polyclonal anti-ERp29 (Abcam ab11420, 1/500) for detecting the ER marker protein, rabbit polyclonal anti-LAMP (Abcam ab24170, 1/100) for detecting the lysosome marker protein, and mouse monoclonal anti-Reelin (Abcam ab78540, 1/400) were used. The cells were incubated overnight at 4 °C with primary antibody with 5 % normal goat serum. The cells were washed five times for 10 min in PBST and then soaked in 5 % normal goat serum in PBST containing corresponding secondary antibodies, goat anti-mouse IgG-Alexa 488 (green, Invitrogen A11029, 1/1,000) and goat anti-rabbit IgG-Alexa 594 (red, Invitrogen A11037, 1/1,000) for 90 min in the dark. Finally, cells were washed five times for 10 min in PBST and three times for 5 min in PBS, then processed for Hoechst 33258 (Sigma B2883) for 30 min, washed in PBS, and cover-slipped with Fluoromount. Immunofluorescent images were visualized using confocal microscope (Olympus FV1000). Control experiments were performed with the omission of the primary antibodies yielding negative results.

### Data Analysis

Data were evaluated using one-way analysis of variance (ANOVA) followed by post hoc Bonferroni’s *t* tests. Statistical significance was preset at *p* < 0.05. Data are represented as means ± SD from quintuplicates of each treatment condition. The value of *IC*_50_ was calculated from the equation *Y* = *A* + [(*B* − *A*) / (1 + (*x* / *IC*_50_)^*h*^) + (*B* − *A*) / (*F* − *A*) − 1], where *Y* is the observed value of individual KA concentration (*x*), *A* is the y axis value of the bottom plateau, *B* is maximal value, *x* is the concentration of KA, *h* is the Hill coefficient (*h* ≈ 1), *F* = (*B* + Baseline) / 2, and Baseline is the *y*-axis value that defines 0 %, maximal inhibition by KA [28]. The *IC*_50_ values were estimated by plotting the logarithmic values of the different KA doses using GraphPad Prism 6® software.

## Results

### Treatment of PRNCs with KA Resembles the Early Stage of Epilepsy

**Fig. 1:**
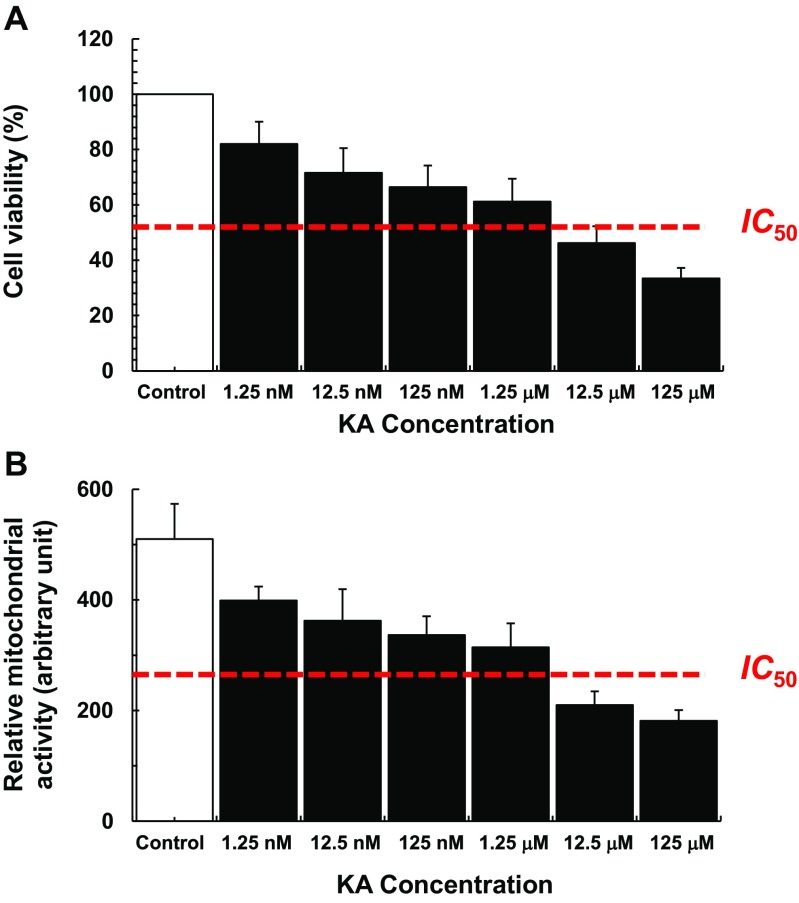
Dose-response effects on cell viability and relative mitochondrial activity based on KA concentrations. After cultured PRNCs were incubated with KA for 3 days at 37 °C, cell viability and mitochondrial activity were measured by using calcein assay (**a**) and MTT assay (**b**), respectively. The *IC*_50_ values were estimated by using GraphPad Prism 6® software

**Fig. 2:**
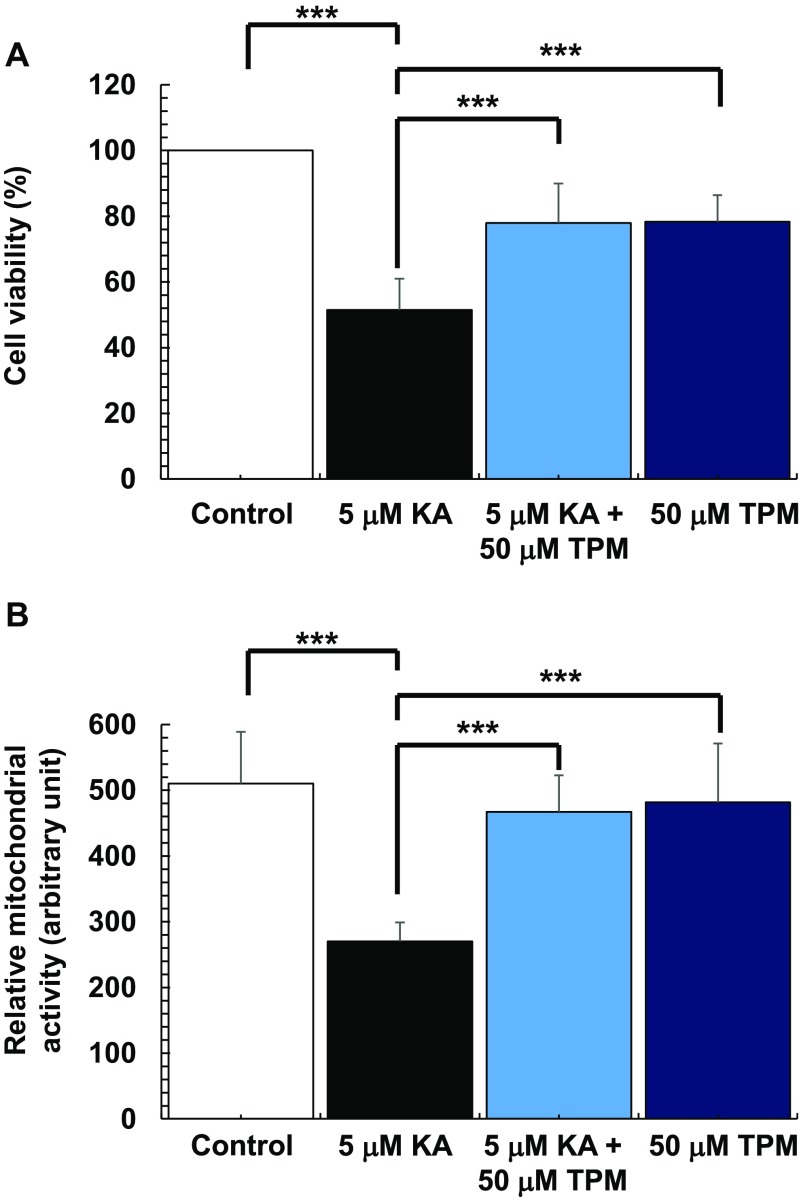
Topiramate (TPM) significantly reduced both cell death and mitochondrial dysfunction induced by KA administration. Cultured PRNCs were subjected to 5 μM KA alone, or 5 μM KA in the presence of 50 μM TPM, or 50 μM TPM alone for 3 days at 37 °C, then cell viability and mitochondrial activity were measured by using calcein assay (**a**) and MTT assay (**b**). There were no significant differences in both cell viability and mitochondrial activity between control and 50 μM TPM only treatment. ***P < 0.001

PRNC viability and mitochondrial activity were significantly decreased in a dose-dependent manner with KA administration; the estimated *IC*_50_ values for cell viability and mitochondrial activity are 5.89 ± 0.185 μM and 2.79 ± 0.515 μM, respectively (Fig. 1). Topiramate [2,3:4,5-bis-*O*-(1-methylethylidene)-*b*-d-fructopyranosesulfamate] (TPM, Sigma-Aldrich T0575), a commonly used AED [21, 22], antagonizes the AMPA/kainate receptor-mediated signaling pathway in cultured neurons in which the drug was shown to produce a slow depression of KA inward current activation [29]. To confirm that reduction of both cell viability and the mitochondrial activity depends on the KA-induced activation of AMPA/kainate receptors, cell viability and the mitochondrial activity were compared in the presence of both KA and TPM with that of KA alone. After exposure to 5 μM KA, both cell viability (*F*_2,26_ = 73.37, *p* < 0.0001) (Fig. 2a) and mitochondrial activity (*F*_2,23_ = 43.87, *p* < 0.0001) (Fig. 2b) were significantly decreased. Treatment of PRNCs with TPM increased both cell viability and mitochondrial activity in PRNCs exposed to KA (*p* < 0.05). Altogether, these results indicate that this in vitro KA paradigm is reminiscent of early stage epilepsy.

### KA Induces the Collapse of ER-Lysosome-Golgi Network

**Fig. 3:**
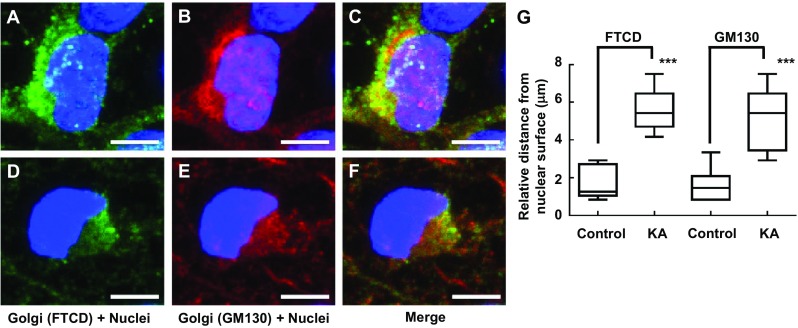
Immunofluorescence showing gross changes in Golgi complex distribution/morphology in response to KA administration with PRNCs for 3 days at 37 °C. PRNCs were fixed and stained for the Golgi complex using antibodies against FTCD (*green*) and GM130 (*red*) and for nuclei using Hoechst 33258 (*blue*). **a**–**c** Control, FTCD, and GM130 work in tandem with localizing closely nuclei, whereas **d**–**f** PRNCs were incubated with 5 μM KA for 3 days at 37 °C. **g** Quantifications correspond to Golgi fragmentation, distance of reconstructed anti-FTCD, and anti-GM130 fluorescent signal from nuclear surface. In control condition, *P* value of FTCD versus GM130 is 0.687, and the value in KA condition is 0.369. KA-induced epilepsy provoked Golgi complex fragmentation/dispersal. ***P < 0.001. *Scale bars* = 5 μm

**Fig. 4:**
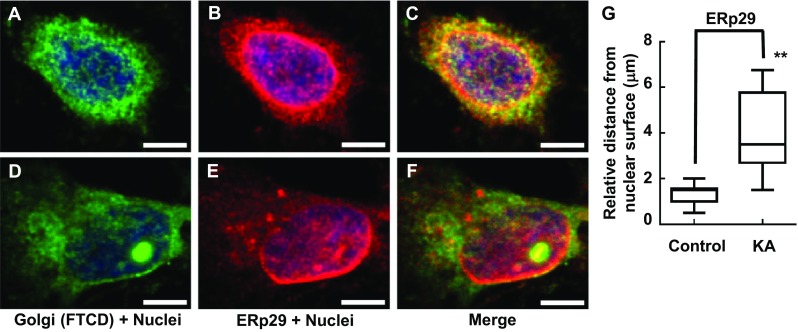
Immunofluorescence showing gross changes interaction between Golgi complex and ER distribution/morphology in response to KA administration with PRNCs for 3 days at 37 °C. PRNCs were fixed and stained for the Golgi complex and ER using antibodies against FTCD (*green*) and ERp29 (*red*), respectively, and for nuclei using Hoechst 33258 (*blue*). **a**–**c** Control, Golgi closely localized with ER which distributed around nuclei. **d**–**f** PRNCs were incubated with 5 μM KA for 3 days at 37 °C. KA-induced epilepsy provoked Golgi complex fragmentation/dispersal, and ER are seen dislocated around nuclei. **g** Quantification of ER dislocation from nuclear surface corresponded to distance of reconstructed anti-ERp29 fluorescent signal from nuclear surface. **P < 0.01. *Scale bars* = 5 μm

**Fig. 5:**
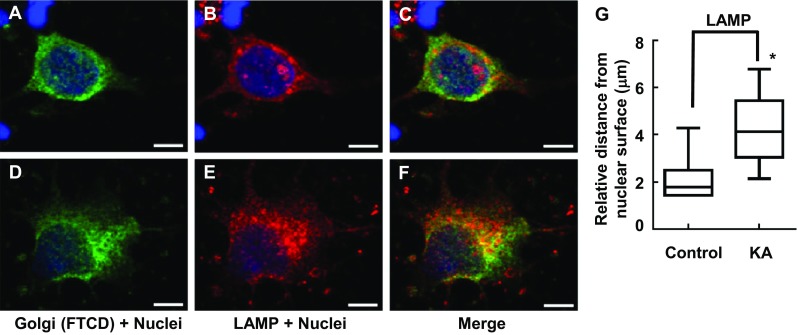
Immunofluorescence showing gross changes interaction between Golgi complex and lysosome distribution/morphology in response to KA administration with PRNCs for 3 days at 37 °C. PRNCs were fixed and stained for the Golgi complex and lysosome using antibodies against FTCD (*green*) and LAMP (*red*), respectively, and for nuclei using Hoechst 33258 (*blue*). **a**–**c** Control, Golgi, and lysosome are closely localized. **d**–**f** PRNCs were incubated with 5 μM KA for 3 days at 37 °C. KA-induced epilepsy provoked Golgi complex and lysosome dispersal. **g** Quantification of lysosome dispersal corresponded to distance of reconstructed anti-LAMP fluorescent signal from nuclear surface. *P < 0.05. *Scale bars* = 5 μm

FTCD co-localizes with GM130 on the Golgi complex membrane [30]; therefore, these antibodies were used in combination to assess Golgi complex structure. Following incubation of PRNCs in 5 μM KA (previously calculated *IC*_50_ of cell viability) for 3 days at 37 °C, Golgi complex fragmentation/dispersal (Fig. 3) and collapse of ER-lysosome-Golgi network were observed (Figs. 4 and 5). This study is the first to report that the Golgi complex fragmentation occurs in the early stages of epilepsy in vitro. The Golgi complex not only serves a pivotal role in trafficking of ion channels, plasma receptors, and other transmitter molecules but also mediates transport of exogenous molecules by retrograde and trans-synaptic signal transduction [4]. Appropriate Golgi complex localization/positioning is essential for the processing, sorting, and transport of proteins and lipids involved in cell wound healing and maintenance of the membrane potential [3]. We hypothesized that Golgi complex fragmentation/dispersal influences the processing of extracellular matrix molecule Reelin.

### Golgi Complex Fragmentation/Dispersal Influences Reelin Processing

**Fig. 6:**
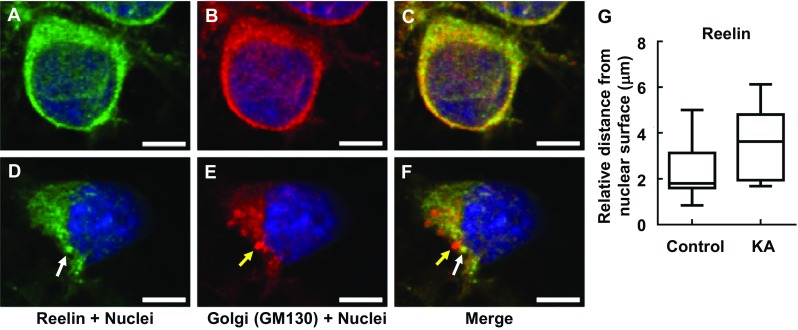
Intracellular Reelin (*white arrow*) dislocated at Golgi complex (*yellow arrow*) in response to KA. PRNCs were cultured with KA for 3 days at 37 °C. PRNCs were fixed and stained for Reelin and Golgi complex using antibodies against Reelin (*green*) and GM 130 (*red*), respectively, and for nuclei using Hoechst 33258 (*blue*). **a**–**c** Control, Intracellular Reelin co-localized with Golgi complex, **d**–**f** PRNCs were incubated with 5 μM KA for 3 days at 37 °C. **g** Quantification of Reelin distribution around nuclei corresponded to distance of reconstructed anti-Reelin fluorescent signal from nuclear surface. *Scale bars* = 5 μm

**Fig. 7:**
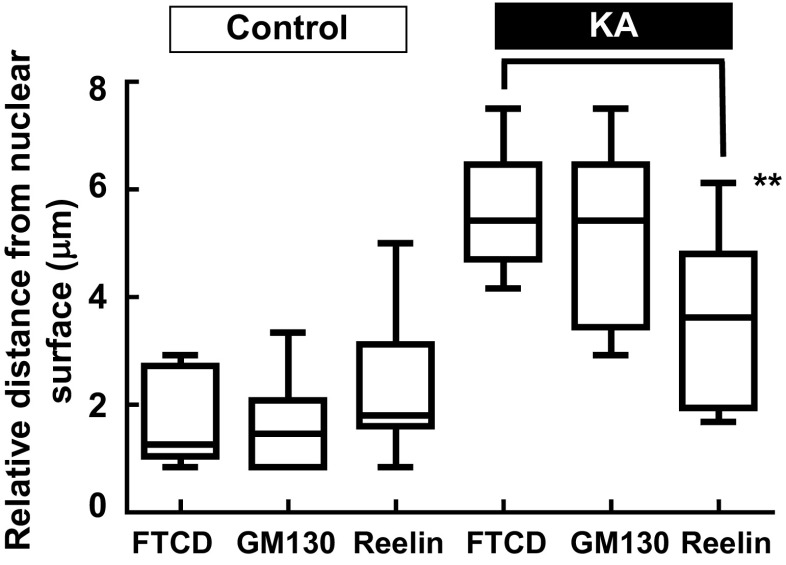
Quantification of intracellular Reelin dislocated from Golgi complex. Comparison of distance from nuclear surface. The distribution of intracellular Reelin consists of Golgi complex in control condition, whereas in KA condition, the division of Reelin separated from distribution of Golgi complex. **P < 0.01

**Fig. 8:**
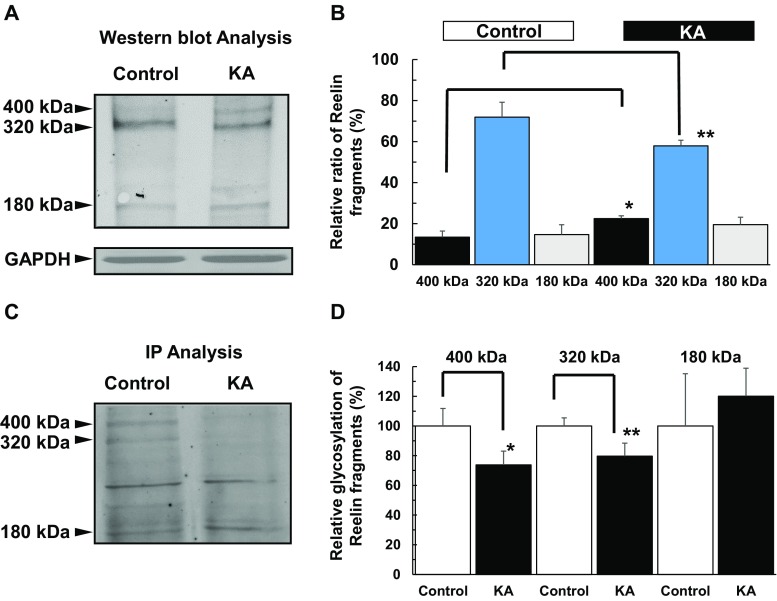
Golgi complex fragmentation impairs Reelin mobilization. **a**, **b** Western blot analysis using anti-Reelin mouse antibody showed Reelin bands (180, 320, and 400 kDa). In healthy neurons (control), bands of 320 and 400 kDa Reelin mobilized shorter than that of epilepsy model (KA). **c**, **d** IP analysis using anti-Reelin mouse antibody demonstrated that the density of 320-kDa Reelin fragment and the full-length Reelin protein (400 kDa) in healthy neuronal condition is significantly higher than that of epilepsy model, although 180-kDa Reelin fragment is similarly expressed in both conditions. *P < 0.05. **P < 0.01

Reelin is an extracellular matrix glycoprotein that has important functions in brain development, adult neurogenesis, and maintenance of synaptic plasticity [12]. Reelin is tightly regulated at ER-lysosome-Golgi network and modified at Golgi complex [13]. Matrix metalloproteinases cleave the full-length Reelin protein (400 kDa) into both 180- and 320-kDa fragments [31]. The 320-kDa fragment is required for proper protein folding and essential of signaling pathways’ activity via the specific Reelin receptors. We found that following KA-induced Golgi complex fragmentation, intracellular Reelin was dispersed from the Golgi complex (Fig. 6, represented by arrow heads and Fig. 7). Of note, there are three types of intracellular Reelin (180, 320, and 400 kDa), which unfortunately could not be distinguished intracellularly via immunocytochemistry when examining these Reelin subtypes and glycosylation. Thus, for immunocytochemistry, while the relative distances from nuclear surface of FTCD, GM130, Erp29, and LAMP were significantly longer in KA than controls (Figs. 3–5), Reelin was detected not to significantly differ from the controls (Fig. 6). On the other hand, protein expression (as shown in subsequent Western blot analysis) clearly showed the changes in ratio of intracellular Reelin subtypes and their glycosylation patterns. Accordingly, Western blot analysis (Fig. 8a, b) revealed that the 320-kDa fragment, representing the secreted form of Reelin[32], was significantly decreased in KA-treated PRNCs compared to control, whereas the full-length Reelin protein (400 kDa) in KA-treated PRNCs was significantly increased (*p* < 0.05), indicating that proteolytic processing of Reelin was decreased in this acute KA neurotoxicity paradigm that mimics the early stage of epilepsy. Furthermore, the mobility of the top two bands in control was slightly shorter than that of PRNCs treated with 5 μM KA for 3 days at 37 °C, demonstrating that these Reelin fragments are more extensively processed under normal physiologic conditions when compared with the KA-induced neurotoxicity in vitro. Accordingly, this KA-induced epilepsy likely contributed to the initiation of Golgi complex fragmentation and the disruption of Reelin protein processing, subsequently resulting in dysfunctions such as decreased secreted Reelin, as well as impaired glycosylation.

**Fig. 9:**
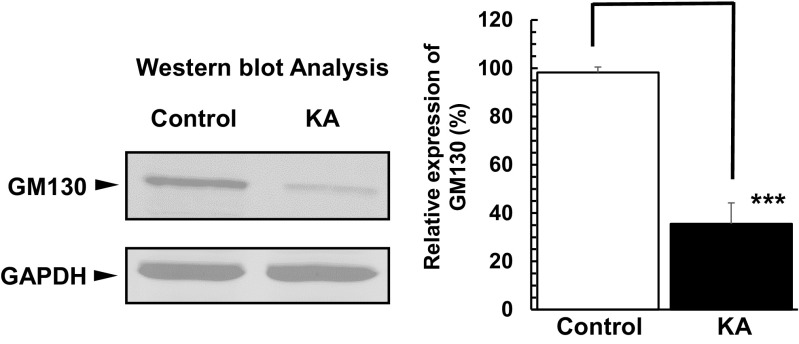
Western blotting analysis of GM130 expression in PRNCs. Following incubation of PRNCs in 5 μM KA for 3 days at 37 °C (KA) or in the absence of KA (control), GM130 expression level under KA condition was significantly decreased, indicating pH acidity of PRNC cytosol. ***P < 0.001

HNK1 glycoepitope is expressed on the extracellular matrix of protein Reelin and is sequentially biosynthesized by glucuronyltransferase and/or sulfotransferase on the Golgi complex membrane [33]. To confirm whether Golgi complex fragmentation affects the glycosylation of Reelin, we performed Western blot analysis using the anti-HNK-1 antibody after Reelin fragments that were immunoprecipitated (described in “Materials and Methods”). We observed higher levels of glycosylated Reelin (320 and 400 kDa) in the control group when compared to our experimental group of PRNCs treated with 5 μM KA for 3 days (Fig. 8c, d), although the glycosylated 180-kDa Reelin fragment was similar between groups. These data demonstrated that Golgi complex fragmentation impairs not only proteolytic processing of Reelin but also its glycosylation in the Golgi apparatus (Fig. 8), resulting in a decrease of physiologically active Reelin in the early stages of epilepsy. Moreover, GM130 co-localizes with FTCD on the Golgi complex membrane and is a sensor of intracellular pH level. Here, we found that GM130 expression level under KA condition was significantly decreased (Fig. 9), suggesting PRNCs cytosol pH became acidic.

## Discussion

Epilepsy may arise from multiple genetic mutations that produce functional deficits in a variety of voltage-dependent and/or ligand receptor-mediated ion channels. For example, dysfunction of a voltage-dependent Na^+^ channel in γ-aminobutyric acid (GABA) interneurons is critically associated with the severe myoclonic epilepsy in infancy [34]. Despite the availability of several AEDs, which antagonize the activity of specific ions channels, greater than 30 % of TLE patients will have seizures that are refractory to all standard therapeutics. The precise mechanisms that contribute to the initiation and propagation of seizure activity have not yet been clearly defined [22]; therefore, this study sought to examine the role by which Golgi complex fragmentation initiates epilepsy pathophysiology in a cell culture paradigm.

Progress in investigating epilepsy has been facilitated by the establishment of in vitro and in vivo epilepsy models [2]. Epilepsy treatment has rationally involved AEDs [22] and subsequently has been explored using surgical approaches including electrical stimulation [35]. TPM restricts excitatory neurotransmission at the postsynaptic membrane, enhances inhibitory neurotransmission by allosterically modulating GABA_A_ receptor-mediated Cl^−^ current, and reduces spontaneous motor seizures in rats with KA-induced epilepsy [21]. A recent study reported that KA administration into the rat brain, resembling a chronic model of epilepsy in vivo, altered the proteolytic processing of Reelin [18]. Within short durations, following administration of KA reduced both cell viability and the mitochondrial activity in a dose-dependent manner (Fig. 1), and TPM significantly reduced KA-induced neural toxicity (Fig. 2), indicating that this paradigm closely mimicked in vitro early stage epilepsy. Our acute KA-induced epilepsy model in neuronal cells allowed detection of Golgi complex fragmentation/dispersal (Figs. 3–6) and the collapse of ER-lysosome-Golgi network (Figs. 4 and 5). In consideration of this previous study [18] indicating Reelin as closely associated with cell death in a chronic model of epilepsy, the present data now implicate that Reelin also may play a critical role in the early stages of epilepsy, as a result of Golgi complex fragmentation. Additional in vivo experiments are warranted to further elucidate the molecular mechanisms accompanying this Reelin-Golgi complex fragmentation in epilepsy models.

Despite being linked to a significant number of neurodegenerative diseases, the role of Golgi complex fragmentation in mediating neurodegeneration remains relatively underexplored [23, 24]. The initiation of Golgi complex fragmentation in Alzheimer’s disease has been reported to involve Cdk5 phosphorylating the pH-sensitive *cis*-Golgi protein GM130, and subsequently, GM130 is digested in lysosome [7]. Degradation of GM130 is associated with decreasing intracellular pH, and its depletion interferes with ER-lysosome-Golgi vascular trafficking [36]. We observed decreased expression of GM130 in the acute stage of KA-induced neurotoxicity in comparison with healthy control groups (Fig. 9). Here, we found that epileptic activity was induced in vitro via KA-mediated activation of glutamatergic kainate receptors that respond to the neurotransmitter glutamate, coupled with Golgi complex fragmentation (Figs. 3–6). Glutamate deactivation occurs predominantly by glutamate transporter (EAA) in synapse and by glutamine synthase which catalyzes glutamate neutralization in adjacent glial cells. Excessive glutamate influx overloads the neutralizing capacity of EAA and hampers glutamate-glutamine recycling activity, leading to decreases in the intracellular pH in neurons. With these observations in mind, excessive activation of glutamate receptors on neurons may cause extreme decrements in intracellular pH, which appears to trigger the initiation of Golgi complex fragmentation and spread of seizure area in an epileptic brain. An alternative possibility is that ROS/lipid peroxides or unexpected secondary products directly attack the Golgi complex membranes leading to disruption of Golgi’s ultra-ribbon structure and its fragmentation.

Protein glycosylation is an important posttranslational process that occurs at the ER-lysosome-Golgi network. Glycosylation serves to stabilize the three-dimensional structure of the protein, allowing for selective protein interactions that permit multiple distinct signal transduction patterns from the same gene. Furthermore, glycosylation aids in protein folding, affording intermediates more hydrophilic, which prevents protein aggregation, a crucial cascade event for neurodegeneration. Indeed, many pathologic proteins associated with neurodegenerative diseases (e.g., amyloid precursor protein, β-secretase 1, tau, α-synuclein, and superoxide dismutase) are glycosylated [37]. Although the molecular mechanisms underlying protein glycosylation disorders are quite complex, it has been reported that interruption of the ER-lysosome-Golgi network contributes to the progression of neurodegenerative diseases. Processing glycosylation of Reelin is tightly regulated in ER/Golgi apparatus. Reelin expression and glycosylation patterns are altered in the frontal cortex and cerebellum of Alzheimer’s disease patients [11]. The 180-kDa Reelin fragment interacts with α3β1 integrin receptors [38] but does not interact with ApoER2 and VLDL receptors [39]. In the present study, protein expression and glycosylation levels of 180-kDa Reelin fragments were comparable between control (healthy neurons) and PRNCs treated with 5 μM KA for 3 days (albeit early stage epilepsy) (Fig. 8). Expression and glycosylation of 320-kDa Reelin fragment were significantly increased in control compared with the KA-treated group, suggesting that Golgi complex fragmentation and collapse of the ER-lysosome-Golgi network likely caused the decreased Reelin processing which might have contributed to initiation and progression of epilepsy. Administration of Reelin into the hippocampus has been shown to enhance LTP as result of the activation of SFKs, which increase *N*-methyl-d-aspartate (NMDA) receptor activity [16]. These observations advance the notion that 320-kDa Reelin fragment protein is a major neuromodulator that (1) increases glutamatergic neurotransmission mediated by the postsynaptic ApoER2 and VLDL receptors and therefore enhances synaptic plasticity, and (2) prevents the suppression of NMDA receptor and LTP in epilepsy patient’s brains and altered expression of Reelin (fragments ratio) and its signal transduction (glycosylation patterns), in part, participate in the initiation of epilepsy.

The present results revealed that Golgi complex fragmentation in initiation of KA neurotoxicity could decrease the physiological function of Reelin. Golgi complex fragmentation and Reelin dysfunction may be considered as disease biomarkers, as well as therapeutic targets for epilepsy. A limitation of the present study is the use of rat E18 primary cortical cultures, with all endpoint readouts performed after 3 days in vitro. Accordingly, at this stage, the neurons were immature and had not formed any functional synaptic contacts yet. In order to recognize whether the observed aberrations in Golgi and Reelin translate to altered functional synaptic connectivity of primary neurons will require the use of mature functionally interconnected neurons. Moreover, we recognized the need for confirming the expression and proper localization of glutamate receptors in primary neurons and complemented by in vivo data to strengthen the clinical relevance of the findings. Given that Reelin is expressed in GABAergic interneurons (representing a minor neuronal population in E18 cortical neurons), additional studies are also warranted to provide some mechanistic insights how KA-induced hyperexcitability in primary neurons alters proteolytic processing and glycosylation of Reelin in interneurons. That epilepsy is a network disease may limit the direct translational application of primary neurons, which represent an artificial system with a specific number of neuronal subtypes. Nonetheless, the present results provide valuable informative data for understanding disease pathology and developing treatments directed at targeting immature neurons expressing Golgi and Reelin dysregulation. In this study, we report that KA irreversibly damages the endoplasmic reticulum-lysosome-Golgi network which then leads to abnormal Reelin proteolytic processing. Restoring and reinforcing the ER-lysosome-Golgi network and its interaction with Reelin could be exploited as novel therapeutic regimens for attenuating epilepsy and relevant neurodegenerative diseases.
